# The effect of different force magnitudes for placement of orthodontic brackets on shear bond strength, in three adhesive systems

**DOI:** 10.4317/jced.54733

**Published:** 2018-06-01

**Authors:** Amir Mohammadi, Sohrab Pourkhameneh, Amir-Houman Sadrhaghighi

**Affiliations:** 1 Orthodontist, Associate Professor, Department of Orthodontics, Faculty of Dentistry, Tabriz University of Medical Sciences, Tabriz, East Azerbaijan, Iran; 2 Orthodontist, Private Practice, Ardabil, Iran; 3 Orthodontist Assistant Professor, Dental Faculty, Tabriz Medical Sciences University, Tabriz, Iran

## Abstract

**Background:**

Amount of pressure exerting on orthodontic brackets during bonding can create different thickness of adhesive and affect shear bonding strength(SBS) in different adhesive systems. The purpose of this study was to evaluate the effect of different force magnitudes for placement of brackets on SBS.

**Material and Methods:**

In an *in vitro* study, 420 brackets were placed on the bovine teeth, using three types of adhesives, Concise (chemically cured two-paste mix), Unite (chemically cured no mix), and Transbond XT( light cured), with the application of seven force magnitudes of 50, 100, 200, 300, 400, 600 and 1000 grams in twenty-one groups of twenty samples each. SBS means (using two-way ANOVA with Tukey’s post-hoc test) and adhesive remnant index were compared between these twenty-one groups.

**Results:**

SBS increased with an increase in force. No increase in Transbond XT SBS happened after 400 grams. In addition, Transbond XT had the lowest bond strength among three adhesives (*p*<0.001). Adhesive remnant index (ARI) results also indicated a shift in the failure mode from bracket-adhesive interface to adhesive-enamel interface, as the bonding force got heavier (*p*<0.05).

**Conclusions:**

The force applied on bracket during bonding influences the SBS. In order to have higher bond strength, application of heavy force would be advisable. It is also recommended that constant forces be applied for bracket bonding in future studies.

** Key words:**Adhesive, ARI, Bonding procedures, SBS, shear bond strength.

## Introduction

Achieving high bond strength of the orthodontic brackets to enamel and a low failure rate are the basic demands for a bracket-bonding system, since replacing loose brackets is inefficient, time-consuming, and costly. Consequently, a continuous search is on for higher bond strengths, better adhesives, simpler procedures, and so forth. A substantive number of studies have been focusing on brackets, adhesive systems, and enamel surface conditioning methods in recent years. However, most bond failures result from inconsistencies in the bonding technique and not because of the bonding resins, the bracket base, or quality of the enamel etching used (1,2).

In order for an appropriate bonding procedure, after transferring and positioning of the brackets, pushing them firmly toward the tooth surfaces is called essential by some papers (2,3). The rationale behind this statement declared as: the tight fit will result in good bond strength, little material to remove on debonding, optimal adhesive penetration into bracket backing, and reduced slide when excess material extrudes peripherally (4). On the other hand applying heavy force on brackets may reduce adhesive thickness and effect on bonding quality is unclear (5).

However, there are few if any studies about the effect of the pressure exerting on brackets during bonding upon shear bond strength, especially among different adhesive systems. The purpose of this study was to evaluate the effect of different force magnitudes for placement of brackets on shear bond strength (SBS), in three adhesive systems.

## Material and Methods

-Teeth

Four-hundred-and-twenty freshly extracted permanent bovine mandibular incisors were collected from a local slaughterhouse (3). To meet the criteria for this study, the teeth were selected only if they had intact buccal enamel and had no surface cracks from extraction forceps. The teeth were cleansed of soft tissue and the roots were embedded vertically in cold-cured, fast setting acrylic (Acropars 200, Marlic Co, Iran) with their crowns exposed, avoiding contact between the resin and crown. A mounting jig was used to align the facial surfaces of the teeth perpendicular with the bottom of the mold. This kept the facial surface of the tooth parallel to the applied force during the shear test. After mounting, the teeth were stored in a solution of 0.2% (weight/volume) thymol (6,7). The teeth were randomly assigned to one of 21 groups.

-Adhesives

Three types of adhesives were tested in this study: chemically cured two-paste mix (Concise, 3M Unitek, Monrovia, California, USA), chemically cured no mix (Unite, 3M Unitek, Monrovia, California, USA) and light cured (Transbond XT, 3M Unitek, Monrovia, California, USA). The pressure exerted on brackets during the bonding was categorized into seven magnitudes of force: 50, 100, 200, 300, 400, 600 and 1000 grams. A total of 420 brackets were placed on the bovine teeth, using three types of adhesives and the application of seven force magnitudes in twenty-one groups of twenty samples each.

A standardized protocol of tooth preparation and bracket bonding was adopted (5). Before bonding, the enamel surfaces were polished with a mixture of water and fluoride-free pumice (Oral-B, Frankfurt a. M., Germany) using a rubber polishing cup for 10 seconds. Thereafter, the enamel surfaces were etched for 30 seconds with the recommended etching liquid supplied by the manufacturer (37% orthophosphoric acid). The teeth were rinsed thoroughly with water for 10 seconds and dried with oil-free compressed air for another 10 seconds. In all cases the frosty white appearance of etched enamel was noticed. The adhesives were applied to the bracket base in accordance with the manufacturers’ instructions. Commercially available maxillary right central incisor metal brackets with 0.018” slots were used (Ortho-organizer, Carlsbad, California, USA).

In each group of adhesives, whenever the sealant/primer were applied on the tooth surfaces, a thin coat was applied to the etched area of the teeth using a nylon brush. The brush was dipped in the primer for each tooth to be primed. Air was gently blown on each tooth for 1-2 seconds, aiming the air stream perpendicular to the enamel surface.

-Bonding procedure

The exact bonding procedure is described below for each group of adhesives, separately:

Concise: A mixture of sealant A + B was first applied on the etched enamel surface. The mixed pastes A + B were then put on the bracket base and the bracket was placed on the sealed tooth surface. After exerting the predetermined force to seat it, excess adhesive surrounding the bracket was gently removed with a scaler.

Unite: A thin coat of the primer (catalyst) was applied to the the enamel surface and bracket base, followed by the adhesive paste application on the bracket base. The bracket was then applied to the enamel surface. After exerting the predetermined force to seat it, excess adhesive surrounding the bracket was gently removed with a scaler.

Transbond XT: A thin coat of the sealant was applied to the the enamel surface, followed by the adhesive paste application on the bracket base. The bracket was then applied to the enamel surface. After exerting the predetermined force to seat it and removal of excess resin, the adhesive was light-cured for 20 seconds from the both proximal margins by a tungsten halogen light unit with 450 nm wavelength and 280 ±5 mW/cm2 (Litex 680A, DentAmerica, City of Industry, California, USA).

The predetermined forces exerted on brackets during the bonding were applied by a universal materials testing machine (Hounsfield Test Equipment, H5KS, Surrey, UK) up to 50, 100, 200, 300, 400, 600 and 1000 grams, respectively. A pointed crosshead with a speed of 0.5 mm/minute was used. Immediately after reaching the predetermined magnitude of bonding force, the pressure on the bracket was automatically removed. The specimens were then stored in distilled water at 37°C for 24 hours before bond strength testing.

-Debonding procedure

After completing the procedures, the embedded specimens were secured in a jig attached to the base plate of a universal materials testing machine (Hounsfield Test Equipment, H5KS, Surrey, UK), so that the bracket base of the sample paralleled the direction of the shear force. A chisel-edge plunger was mounted in the movable crosshead of the testing machine and positioned so that the leading edge was aimed at the enamel/adhesive interface. The specimens were stressed in a gingivo-incisal direction. A crosshead speed of 0.5 mm/minute was used, and the maximum load necessary to debond the bracket was recorded as SBS in megapascal (MPa). The average surface area of the bracket base was determined to be 12.17 mm2 by measuring ten brackets.

-Residual adhesive

After debonding, all teeth and brackets were examined under ×10 magnification (Stereoscopic zoom microscope, Nikon corporation, Tokyo, Japan). The amount of adhesive remaining on the enamel surface was coded using adhesive remnant index (ARI) (8):

0 = no adhesive remains on the tooth surface

1 = less than half the adhesive remains on the tooth surface

2 = more than half the adhesive remains on the tooth surface

3 = all the adhesive remains on the tooth surface.

-Statistical methods

All statistical analyses were performed with the Statistical Package for Social Sciences (SPSS for Windows 13.0; SPSS, Chicago, Illinois, USA). Descriptive statistics including the mean and standard deviation were calculated. Shapiro-Wilks normality test was applied to the SBS data. The data showed normal distribution. Two-way ANOVA with the Tukey’s post-hoc test was used to compare the SBS data. Fracture modes were analyzed using a Pearson’s chi-square test. For the purpose of chi-square test validity, the ARI scores 0, 1 and 2 were combined. Significance was predetermined at *P* < 0.05.

## Results

-Shear Bond Strength

**Figure 1:**
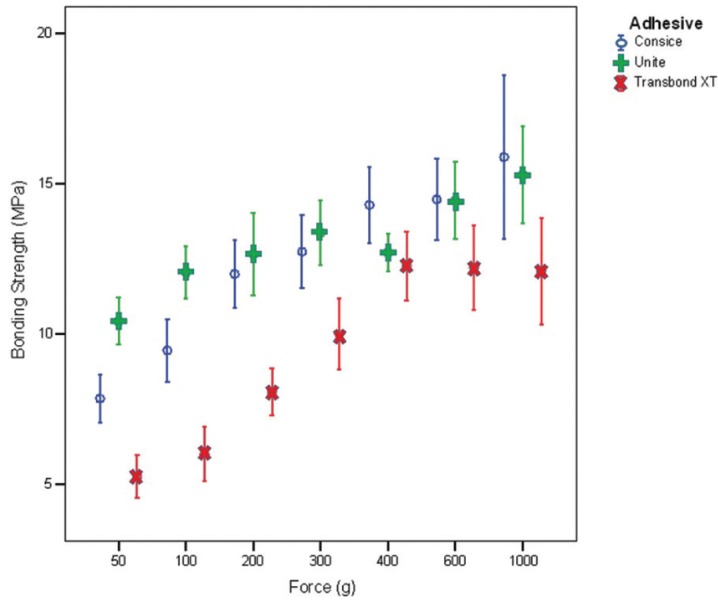
Shear bond strength comparison between three types of adhesives and seven magnitudes of bonding force.

The descriptive statistics for each group tested is summarized in  Table 1 and Fig. 1. The results of two-way ANOVA and Tukey’s post-hoc test are shown in  Table 2 and  Table 3, respectively. The results revealed that the shear bond strength was significantly affected by the type of adhesive and the amount of bonding force. SBS increased with an increase in force. Although, this trend slow down as it approached heavy bonding forces for all adhesives and also no increase in Transbond XT shear bond happened after 400 grams.

**Table 1:**
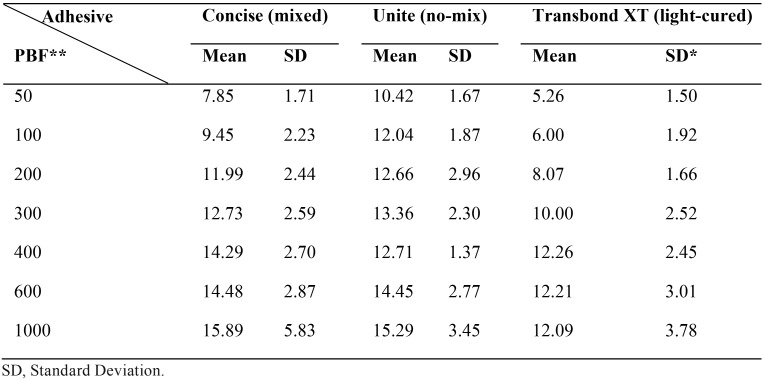
Descriptive Statistics of shear bond test (MPa) for three types of adhesives and seven magnitudes of bonding force.

**Table 2:**
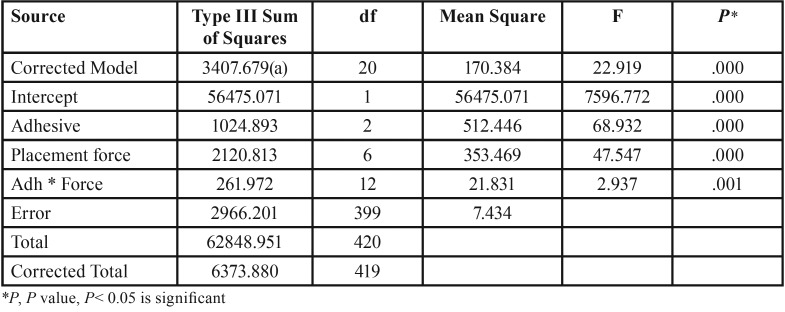
Shear bond strength comparison for three types of adhesives and seven magnitudes of bonding force by two-way ANOVA test.

**Table 3:**
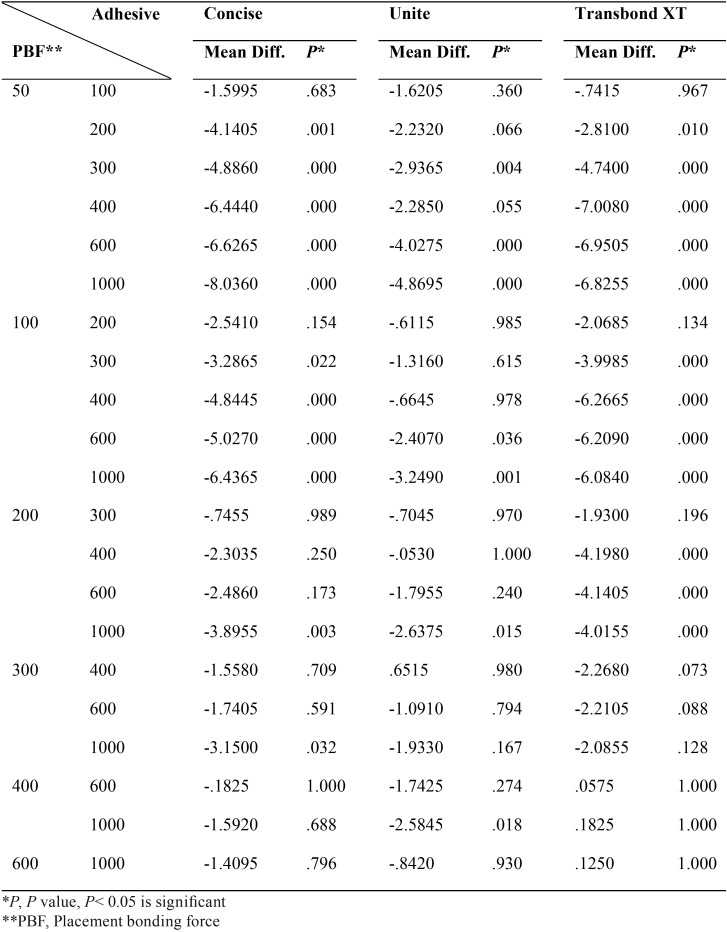
Shear bond strength comparison for three types of adhesives and seven magnitudes of bonding placement force by Tukey’s post-hoc test.

In addition, Transbond XT had the lowest bond strength among three adhesives. It started with the bond strength beneath 6 MPa by 50 g bonding force and reached a plateau above 12 MPa. However Concise had lower bond strength than Unite at first, its bonding strength overtook Unite in heavy bonding forces. Unite showed high bonding strength with almost any bonding forces. Both Concise and Unite achieved nearly 15 MPa by 1000 g bonding force. Shear bond strength of Concise is lower than Unite until 400 grams bonding force. 

-Adhesive Remnant Index

**Figure 2:**
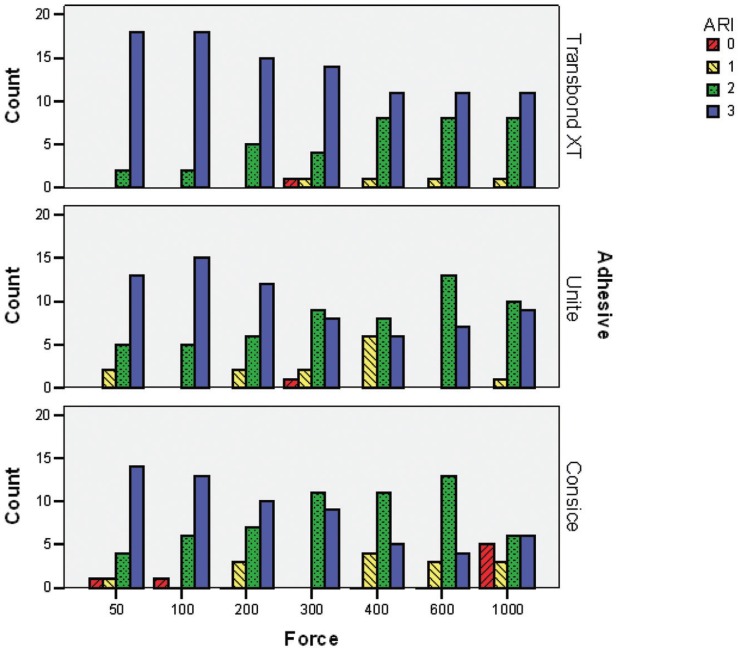
Frequency distribution of the adhesive remnant index (ARI) scores for three types of adhesives and seven magnitudes of bonding force.

The failure modes are presented in Figure 2. After combining ARI scores 0, 1 and 2 the chi-square test indicated that the seven magnitudes of bonding force had significantly different failure modes in each adhesive group (*P* = .005, .034 and .027 for Concise, Unite and Transbond XT, respectively). The failure modes shifted from bracket-adhesive interface to the adhesive-enamel interface, as the bonding force got heavier.

## Discussion

Many articles in restorative dentistry investigated the effect of adhesive thickness on the bond strength of many adhesion systems, especially resin-dentin bond strength. Despite controversial results among these studies, the general consensus suggests that adhesive thickness influences dentin bond strength. Adhesive systems have different viscosities and spread differently onto a substrate, influencing the adhesive layer thickness and the bond strength also. Most of these studies concluded that the bond strength decreased with increasing of the adhesive layer thickness (9-12).

In orthodontics, a few investigations studied the effect of adhesive layer thickness on the bond strength between bracket and tooth surface. Evans and Powers declared in their study that there was a decrease in tensile bond strength as adhesive thickness increased. In Concise, the two-paste adhesive system tested, decreasing of bonding strength was gradual, however the other three no-mix adhesives had a sudden decrease beyond a particular thickness (13).

In the study of Mackay *et al.*, it was shown that each adhesive had its own minimum thickness, probably related to its viscosity. Increasing the thickness of the adhesiveness to 0.26 mm, using a stainless steel spacer had minimal effect on their mean SBS (14).

Jost-Brinkmann *et al.* investigated the influence of varying adhesive layer thickness from a minimum of 0.0 mm to a maximum of 0.8 mm, between bracket base and enamel surface on tensile bond strength. Eight different orthodontic adhesives were investigated. It was found that the highly filled adhesives, such as Concise, provided greatest bond strength and increasing the thickness of the adhesive layer had no significant influence on tensile bond strength. In the case of light-cured adhesives, maximum tensile bond strength was achieved at a thickness of the adhesive layer of 0.2 mm. This is probably due to better penetration of light at this thickness. In the case of chemically curing no-mix adhesives, it was impossible to produce effective adhesive layers thicker than 0.2 mm, presumably because curing at the primer-paste interface becomes a problem at greater thickness. If adhesive layer thickness of more than 0.2 mm is required, a chemically cured, highly filled paste-paste system might be suggested (4).

Knox *et al.* evaluated the influence of orthodontic adhesive thickness on the stresses generated in a bonded bracket using finite element model. Increased stresses were recorded at the lute periphery as the lute dimensions increased. The thickness of the adhesive lute contribute to the stress distribution within the bracket-adhesive-tooth and, therefore, the quality of orthodontic attachment provided (15).

Arici *et al.* reported that the light-cured, resin-modified glass-ionomer adhesive, Fuji Ortho LC, had its highest mean bond strength at the 0.25 mm thickness in both tensile and shear test modes. Although mean tensile bond strengths decreased, mean SBS of Transbond, as the control group, progressively increased when the adhesive thickness increased from 0 to 0.5 mm (16).

In the present study, SBS of all three adhesives increased with an increase in bonding force. Possible reason could be decreased adhesive layer thickness by increasing bonding force, according to the aforementioned literature. Decreasing adhesive layer thickness might give rise to any of the following events:

1) More effective penetration of adhesive into the bracket base.

2) Closer fitness of bracket and tooth surface.

3) Less polymerization shrinkage in smaller amount of adhesive.

4) Fewer trapped air and imperfections (voids and cracks).

5) Deeper cure at the primer-paste interface in no-mix adhesives.

Slowdown of this strengthening pattern could be related to the fact that each adhesive resists against more pressure above a certain thickness. It is probably associated to the consistency and the viscosity of adhesives. This is in agreement with the result presented by Mackay (14).

Transbond showed lower bonding strength by lighter bonding forces, in comparison with Concise and Unite. The role of consistency is possible again, because this adhesive has more consistency than the other two adhesives and it could resist more against pressure and thinning. On the other hand, Unite is less consistent and pressed enough to give higher bond strength.

The failure modes were shifted from bracket-adhesive interface to the adhesive-enamel interface as bonding force increased. It took place due to greater adhesive penetration into the bracket base. This made bracket-adhesive interface stronger, therefore failure occurred in weaker areas. Beside the benefit of stronger bonding strength, failure near the enamel surface could damage it (17). It would be wise to reach strong bonding strength which has little hazardous impact on the enamel.

## Conclusions

1) SBS increases with an increase in bonding force. Considering the influence of bonding force on the bond strength, it is recommended that standard forces are applied for bracket bonding in the studies. Determining of optimum force for bracket bonding with a particular adhesive could be helpful in both the studies and clinical practice.

2) There is a relationship between consistency of the adhesive, film thickness, the force applied during the bonding, and the bond strength.

3) The failure modes are shifted from bracket-adhesive interface to the adhesive-enamel interface with an increase of bonding force.
